# Medical Ethics

**Published:** 1893-11

**Authors:** L. A. Grizzard

**Affiliations:** President of the Taylor County Medical Society; Abilene


### MEDICAL ETHICS.

[Extracts from an address by Dr. L. A. Grizzard, as President of the Taylor County Medical Society.]

IS THE Code of Medical Ethics a humbug? Or are we the humbugs? None of us live up to its principles fully. We are human beings and from the standpoint of weakness, shortcoming and imperfection, that means much.

Shall we live and act so as to raise to a high and more honorable plane our profession, or shall we tear down all restraint, remove all restriction and make medicine a trade?

Better that we declare non-allegience to all that is ethical and honorable, using the words ethical and honorable as synonyms, than *pretend* to be professional, pretend to be supporters of professional ethics, but in reality supporters only with "lip service."

Honesty is just as requisite in the practice of medicine as in any other vocation. If a man is not an honest physician he cannot be an honest man. He cannot long deceive the public, and the profession he cannot deceive at all. Our boastings, our illogical, unethical and *untruthful* harangues with the laity, all reach the ears of the profession sooner or later.

It is a patent fact that we are not to the world what we pretend to be,—but just what we really are. We may profess right things, ethical things with deceptive lips, but we deceive nobody. We may loudly declare our loyalty to the code of ethics of our profession, and express a desire to see organized medicine lifted upon a higher plane of professional honor and dignity, while at the same time we are resorting to all sorts of questionable methods to secure practice,—to persuade families away from their medical attendant,—endeavoring to imbue the people with an idea of our superiority over other medical men.

We need more true manhood, more integrity that dares to do right for right's sake: more thinkers and fewer guessers; more knowledge and less pretension; more real diagnostic power and less flippant assertion; more investigators and fewer pretenders in the practice of medicine.

To be an honest man in the practice of medicine, is no easy matter. Ordinary loose-jointed conceptions of right and wrong are not sufficient to withstand the temptations to be a quack, a charlatan, a slick-tongued mountebank.

The quackery of the traveling specialist is not the greatest enemy of legitimate medicine, but the covert oily-tongued gents who are within the fold. These men are not only a disgrace to the name they bear, but a deadly nightshade to organized medicine. * * * * *

To do unto other physicians, under all circumstances, as we would have them do unto us, is easy to assert. But do we do it? Do we depend upon merit alone to secure practice? Do we resort to questionable practices? Is not "self" *first*, even though it be at the expense of a professional brother. * * * *Why not* be ethical, honest, honorable medical men and secure practice only through legitimate channels?

Let our acts, deeds and words be open and above board, and rid ourselves of the miserable and unmanly practice of *soliciting* patronage. * * * *

I fancy when we have grown old in the practice,—as we stand down the western declivity,—as we near the sunset of life, and retrospectively view our past lives, our medical pilgrimage on earth; when we recall our failures and successes, our ups and downs—that a tear of regret will trickle down our time-furrowed cheeks, when we are reminded of the many bitter things said, unjust criticisms and deceptions practiced.

We are all human, and human nature is weak; but let us live as near as possible up to the spirit as well as the letter of the medical code.

What a happy influence could pervade and permeate every corner of the State, if the physicians of Texas would all co-operate, harmonize, go hand in hand in sustaining the honor and dignity of the healing art! What a wholesome fruitage could each city, village and hamlet enjoy, if the medical fraternity, each one could be depended upon, and every one were truly an ethical physician! How much fraternity, harmony, and happiness would, as a natural sequence, follow.

Then let us be men and not things; let us live up to the requirements of the code, or be man enough to repudiate it in *toto*, and absolve ourselves from the imputation of hypocrisy and charlatanism. Let us be true, ethical physicians, and if, upon our tombstones can be truly incribed: "He was an honest physician," we will not have lived in vain.

